# Impact of IL-2 on Treatment Tolerance in Patients With High-Risk Neuroblastoma Treated With Dinutuximab Beta-Based Immunotherapy

**DOI:** 10.3389/fped.2020.582820

**Published:** 2020-12-16

**Authors:** Filiz Cicek, Sascha Troschke-Meurer, Kiraz Ceylan, Luciana J. Jahns, Maxi Zumpe, Nikolai Siebert, Karoline Ehlert, Holger N. Lode

**Affiliations:** Department of Pediatric Hematology and Oncology, University Medicine Greifswald, Greifswald, Germany

**Keywords:** dinutuximab beta, immunotherapy, interleukin 2, neuroblastoma, toxicity, treatment tolerance

## Abstract

Patients with high-risk neuroblastoma treated with continuous long-term infusion of anti-GD2 antibody dinutuximab beta (DB) in combination with IL-2 show an acceptable safety profile. Here, we compared treatment tolerance with and without IL-2. Ninety-nine patients with high-risk neuroblastoma received up to five cycles of DB given as long-term infusion (10 mg/m^2^/d, 100 mg/m^2^; per cycle) with IL-2 (53 patients; regimen A; 6 × 10^6^ IU/m^2^/d; 60 × 10^6^ IU/m^2^/cycle) and without IL-2 (46 patients; regimen B) in a single-center compassionate use program. Clinical parameters (body temperature, vital signs, Lansky performance score), laboratory values [C-reactive protein, IFN-γ, IL-6, and IL-18 (cycle 1)], and requirement of i.v. co-medication (e.g., morphine, metamizole) were systematically assessed. Patients with stable clinical parameters and that did not require co-medication were defined as potential “outpatient candidates.” Patients showed higher levels of body temperature and CRP in regimen A compared to B. However, IL-6 serum concentrations were similar in pts of both cohorts in the first cycle. Patients receiving regimen B showed a shorter time to achieve normal vital parameters and required less co-medication compared to patients in regimen A that resulted in a shorter median time period to discharge and to achieve a potential outpatient status (6d regimen A and 3–5d regimen B after start of antibody infusion, respectively). This study shows that omitting IL-2 from immunotherapy with DB allows reduced co-medication and hospitalization time and therefore results in improved quality of life in patients with high-risk neuroblastoma.

## Introduction

Neuroblastoma (NB) is the most frequent extracranial solid tumor of childhood and presents with high-risk disease in 50% of cases at initial diagnosis. Treatment consists of a multimodal treatment approach including high-intensity chemotherapy, surgery, radiation, high-dose chemotherapy, and stem cell rescue followed by maintenance therapy. The introduction of anti-GD2-based immunotherapies in combination with interleukin 2 (IL-2) into the maintenance phase prolonged the 5-year event-free survival of patients (pts) with high-risk NB from 50 to 64% (1).

In Europe, the anti-GD2 antibody (Ab) ch14.18 was produced in Chinese hamster ovary (CHO) cells (ch14.18/CHO, dinutuximab beta, DB) and its safety was evaluated in a Phase 1 study (2), where DB was given as short-term intravenous infusion (20 mg/m^2^/d 8 h; 5 consecutive days; cumulative dose: 100 mg/m^2^ per cycle). The safety profile of DB compared to ch14.18 produced in SP2/0 cells (dinutuximab) was not different, and clinical activity was observed (2). This enabled a clinical development program for DB which led to the approval of DB in the European Union for the treatment of high-risk NB in 2017.

The treatment of pts with DB, in particular in combination with IL-2, is associated with neuropathic pain and inflammatory side effects that are managed by co-medication with intravenous analgesics and antipyretics such as morphine and metamizole (3–5). To mitigate these side effects, the DB infusion time was prolonged to 10 days and given as a continuous (24 h) intravenous long-term infusion (LTI of 100 mg/m^2^/cycle, 5 cycles) in combination with IL-2 and oral isotretinoin. Pain intensity was significantly reduced with this application method (6). We previously reported an improved treatment tolerance in pts treated with a LTI of DB in combination with IL-2 that allowed outpatient care (7). The role of IL-2 for the efficacy of DB in NB patients treated with DB given as LTI is still subject of ongoing clinical trials. However, there is no evidence that addition of IL-2 to immunotherapy with DB, given as a short-term infusion (STI), improved outcome in patients with high-risk NB, but was associated with higher toxicity (8). Furthermore, IL-2 preferentially induces regulatory T cells that are associated with poor clinical outcome (9).

To further assess the role of IL-2 in treatment tolerance in the LTI regimen, we investigated clinical parameters, laboratory values, and co-medication in pts treated with DB either in combination (regimen A) or alone (regimen B).

## Patients and Methods

### Patient Inclusion Criteria and Treatment

**Figure 1:**
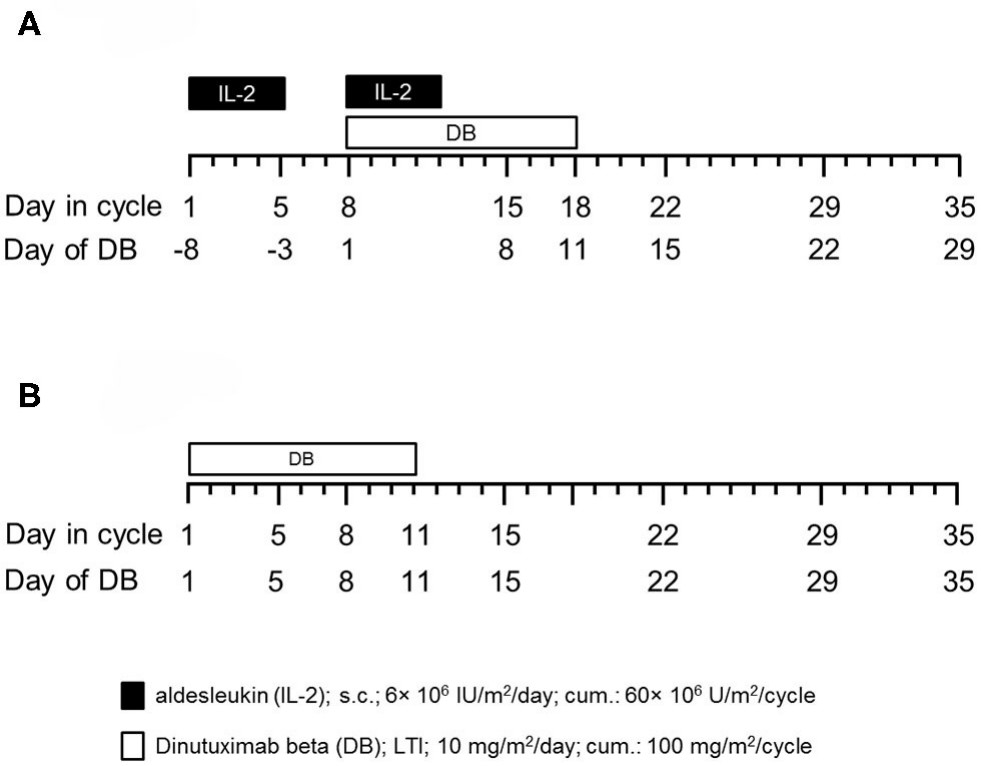
Schematic overview of the treatment schedules. **(A)** Fifty-three patients (pts) with high-risk NB received up to five treatment cycles (35d/cycle) of anti-GD2-based immunotherapy with DB according to the following treatment protocol: IL-2 (aldesleukin; black horizontal bar) was given once a day for 5 days (d1–5, 6 × 10^6^ IU/m^2^/day) followed by combined treatment of i.v. DB administered by long-term infusion (d8–18, 10 mg/m^2^/d; blue horizontal bar) and s.c. IL-2 (d8–12, 6 × 10^6^ IU/m^2^/d). Starting on d19, treatment was continued with oral isotretinoin given twice a day (b.i.d) (d19–32). Cumulative doses of IL-2, DB, and isotretinoin were 60 × 10^6^ IU/m^2^, 100 mg/m^2^, and 2,240 mg/m^2^ per cycle, respectively (regimen A). **(B)** Forty-six pts with high-risk NB were enrolled in a closed single-center compassionate use program and received up to five treatment cycles of DB (35 d/cycle) delivered as a continuous long-term infusion (d1–11, 10 mg/m^2^/d; white horizontal bar). Cumulative dose of DB was 100 mg/m^2^ per cycle (regimen B).

Prior to the immunotherapy, all pts received induction chemotherapy followed by high-dose chemotherapy and autologous stem cell rescue. From 2009 to 2013, 53 pts with high-risk NB, according to the INSS criteria (6), and primary refractory disease (≥2 lines of conventional treatment) (10) were treated with up to five cycles of immunotherapy in a compassionate use program approved by the ethical committee at the University Medicine of Greifswald. One cycle consisted of a LTI of DB (10d of 10 mg/m^2^/day) (Figure 1) with subcutaneous IL-2 (6 × 10^6^ IU/m^2^/day s.c. IL-2) (Figure 1). Due to preliminary results of a large randomized trial (HR-NBL1/SIOPEN) showing that immunotherapy pts did not benefit from the IL-2 treatment, 46 high-risk pts with primary refractory received from 2014 to 2017 the DB-LTI without IL-2 (regimen B) (Figure 1).

The data of pts that discontinued the treatment mainly due to infection or disease progression were excluded from the analysis. Two hundred and twelve cycles were evaluable of the pts that received DB and subcutaneous (s.c.) IL-2 and distributed as follows: In cycle, 1 53 pts and 42 cycles were evaluable. In cycle 2 52 pts and 45 cycles, in cycle 3 50 pts and 43 cycle, in cycle 4 41 pts and 38 cycles, and in cycle 5 38 pts and 35 cycles were evaluable. In pts treated with DB alone, 183 cycles were evaluable: In cycle 1, 46 pts and 36 cycles were evaluable. In cycle 2 44 pts and 41, in cycle 3 41 pts and 37, in cycle 4 39 pts and 36 cycles, and in cycle 5 36 pts and 33 cycles were evaluable.

### Assessment of Treatment Tolerance

Treatment tolerance was analyzed as previously reported (7). Briefly, we daily evaluated a set of clinical assessment parameters such as body temperature, vital signs, Lansky performance status, and requirement of i.v. concomitant medication in the phase of DB therapy for each patient (pt) and cycle. Clinical parameters and the information about co- medication were collected from the pts source data.

DB-LTI (e.g., fever or neuropathic pain) requires hospitalization on d1 of the DB treatment in each cycle. A body temperature ≥ 38.0°C in a 24-h period was defined as fever (11). The criterion “Stable vital signs” included the parameters blood pressure, heart rate, breathing rate, an oxygen saturation value at room air (>95%) was defined as normal. “Normal general health condition” was defined as a Lansky Score above 70% (12). All pts started co-medication prior to the DB LTI including i.v. morphine (cycle 1: 30 μg/kg/h, d1 as long as needed; cycle ≥ 2 as needed), i.v. metamizole (all cycles: 80 mg/kg/d continuous d1 or longer as needed), and oral gabapentin (all cycles: 10 mg/kg/d or longer as needed). The time point when pts did not receive morphine, metamizole or other i.v. concomitant medication (e.g., ondansetron, omeprazole/pantoprazole, dimetindene, dimenhydrinate, or paracetamol) was defined as time until co-medication could be given in an outpatient setting. Pts with all clinical parameters within normal range were defined as “outpatient candidates” that potentially did not require hospitalization.

### Determination of CRP Serum Concentration

CRP serum concentrations (mg/l) were analyzed as a parameter of inflammatory response on d1, d3, d5, d8, and d11 of DB LTI in each treatment cycle by a certified clinical chemistry laboratory at the University Medicine Greifswald.

### Analysis of Cytokine Serum Concentrations

We evaluated serum concentrations (pg/ml) of IFN-γ, IL-6, and IL-18 on days 1, 3, 5, and 8 of DB infusion in each regimen. For that, we used a bead-based immunoassay according to the manufacturer's protocol (Biolegend, cat.nr. 740118).

### Statistical Analysis

For statistical analysis, SPSS (v24, IBM) was used. Data are reported as mean values ± SEM. The difference between two normally distributed independent groups was assessed using the *t*-test. Data with non-parametric distribution were statistically analyzed with the Mann–Whitney *U*-test. Parametric and non-parametric dependent samples were statistically analyzed using Wilcoxon signed-rank test and paired *t*-test, respectively. A *P*-value of < 0.05 was considered significant, < 0.01 very significant, and <0.001 highly significant. Boxplots illustrate median and first and third quartiles, and the whiskers represent values which correspond to 1.5 times the height of the box indicating variability outside the upper and lower quartiles. The closed circles represent outliers that were 3-fold higher than the interquartile range.

## Results

### Comparison of Inflammatory Parameters in Pts Treated With DB With or Without IL-2

The inflammatory response to the treatment was evaluated in 99 pts with high-risk NB (10) receiving either DB plus subcutaneous IL-2 (regimen A) or DB alone (regimen B; Figure 1). The time points refer to days of DB infusion within the respective regimen. We analyzed the body temperature (°C) on days 1–6; CRP (mg/l) on d1, d3, d5, d8, and d11; and serum concentrations of IFN-γ, IL-6, and IL-18 on days 1, 3, 5, and 8 (pg/ml).

**Figure 2:**
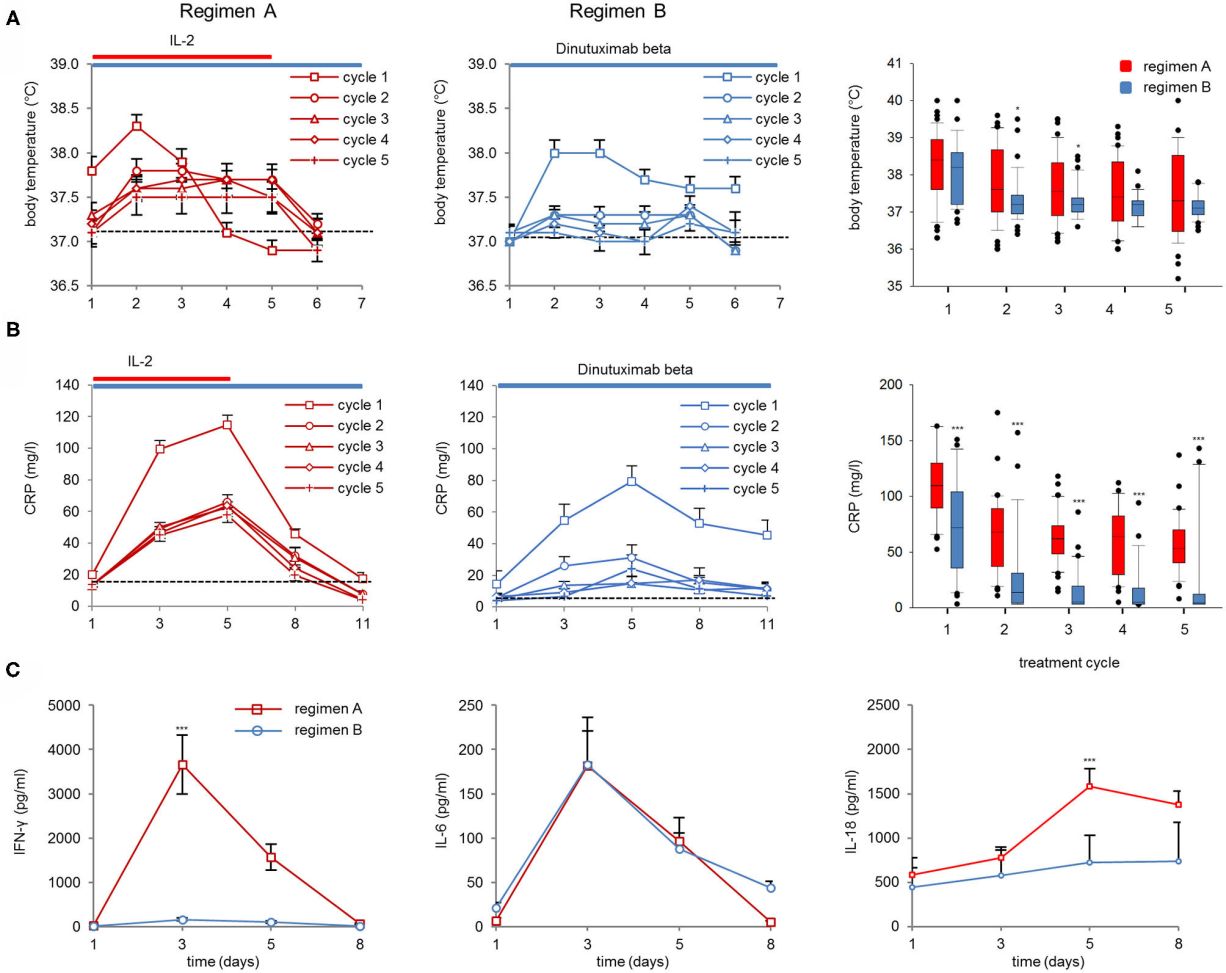
Body temperature, CRP values, and cytokine serum concentrations (IFN-γ, IL-6, and IL-18). **(A)** Body temperature was assessed on the first 6 days of Ab infusion in DB+IL-2 (regimen A) (left panel) or DB pts (regimen B) (center) in cycle 1 (squares), cycle 2 (circles), cycle 3 (triangles), cycle 4 (diamonds), and cycle 5 (crosses). Boxplots of peak body temperature on d2 of Ab infusion in each cycle in DB+IL-2 (red) and DB pts (blue, right panel). **(B)** CRP values were evaluated prior to (d1) during (d3, d8), and at the end of Ab-infusion (d11). Boxplots of CRP values on day of peak levels (d5 of Ab infusion) in each cycle (right panel). DB LTI of the 100-mg/m^2^ treatment is indicated with blue and the delivery of 6 × 10^6^ units/m^2^/day IL-2 delivery with red horizontal lines. **(C)** Cytokine serum concentrations of IFN-γ (left panel), IL-6 (center), and IL-18 (right panel) in pts treated with DB+IL-2 (red, solid line) or DB (blue, solid line). Numbers of pts evaluable for the analysis are described in section Patients and Methods. Data are presented as mean values ± SEM. Statistical analysis was performed using the Mann–Whitney-*U*-test. (**A**, right panel) Significant difference between peak body temperature of DB and DB+IL-2 in cycles 2 and 3 (*P = 0.046 and 0.047, respectively). (**B**, right panel) ***P < 0.001 for cycles 1–5 vs. DB+IL-2. **(C)** *P* < 0.001 vs. DB+IL-2 for IFN-γ and IL-18 on d 3 and 5, respectively.

We observed an increase in body temperature in cycle 1 with an average maximum temperature after 2d of DB either with or without IL-2 (38.3 ± 0.13°C and 37.8 ± 0.13°C, respectively; Figure 2A, left and center panel). In the following cycles, only pts of treatment regimen A showed a body temperature increase above the baseline at 37°C (Figure 2A, right panel). In the following cycles, the peak body temperature per cycle was significantly higher in cycles 2 and 3 during regimen A compared to B (Figure 2A, right panel).

**Figure 3:**
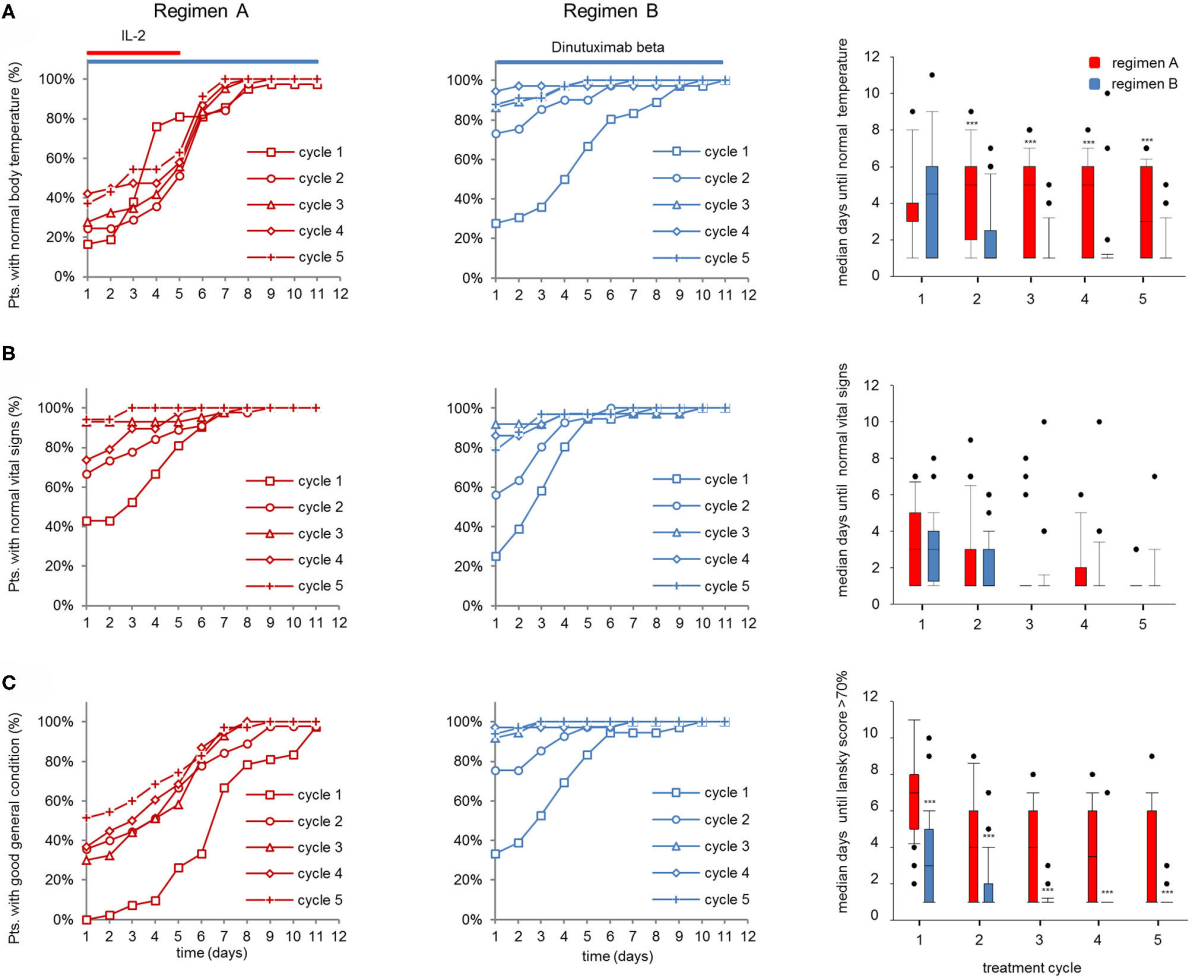
Vital parameters of patients (pts) treated with dinutuximab beta (DB) with or without s.c. IL-2. **(A)** Cumulative number of pts with normal body temperature (<38°C) during the DB+IL-2 (regimen A) (*n* = 53; left panel) and DB treatment (regimen B) (*n* = 46; center) in cycle 1 (squares), cycle 2 (circles), cycle 3 (triangles), cycle 4 (diamonds), and cycle 5 (crosses) in % of the entire cohort. **(B)** Cumulative number of pts presenting stable vital signs (as described in section Patients and Methods) and **(C)** good general health condition (Lansky score > 70%) during the treatment with DB+IL-2 (left panel) or DB (center) in cycles 1–5 in % of entire cohorts. Comparison of median time to achieve normal body temperature, stable vital signs, and Lansky score > 70% is displayed with boxplots (right panel; **A–C**, respectively). DB LTI of 100 mg/m^2^ (10d) treatment is indicated with blue and the delivery of 6 × 10^6^ units/m^2^/day IL-2 with red horizontal lines. (**A–C**, right panels) Comparison of median days until normal body temperature, stable vital signs, and Lansky score > 70% was achieved is shown as boxplots. Statistical analysis was performed using the Mann–Whitney-*U*-test. (**A**, right panel) ***P < 0.001 for cycles 2–5 vs. DB+IL-2. **(C)** ***P < 0.001 vs. DB+IL-2 for cycles 1–5.

The maximum CRP values were found on day five of DB in cycle 1 (2d after maximum body temperature was observed) in both regimens. The peak level was about 2-fold higher in regimen A compared to B (114.9 ± 5.9 and 79.5 ± 9.6 mg/l, respectively; Figure 2B, left and center panel). In regimen A, the peak CRP serum concentrations were significantly higher in all five cycles compared to regimen B (Figure 2B, right panel). Furthermore, we found significantly higher IFN-γ- and IL-18 peak serum concentrations in pts treated with IL-2 compared to those treated only with the Ab (3656 ± 655 and 445 ± 156 pg/ml on day 3 for IFN-γ and 1584 ± 196 and 726 ± 305 pg/ml on d5 for IL-18, respectively; Figure 3C left and right panel). Interestingly, IL-6 serum concentrations were not different between both regimens on d3 of DB infusion (182 ± 55 and 183 ± 38 pg/ml, respectively; Figure 2C, center panel), indicating an Ab-related induction of IL-6 without further increase by IL-2. Finally, all inflammatory parameters, except IL-18, dropped almost to the baseline already during or at the end of DB infusion.

In summary, we found a similar inflammatory response during the first cycle with both regimens that was reduced in pts receiving regimen B in the following cycles compared to regimen A.

### Impact of IL-2 on Vital Parameters During DB Infusion

Next, we determined the impact of the DB LTI with and without IL-2 on the time to achieve stable clinical parameters. Vital parameters, such as body temperature (Figure 3A), stable vital signs (Figure 3B), and Lansky score (Figure 3C), were assessed daily during DB LTI (d1-d11). Median days to achieve stable vital parameters were compared between the groups.

In line with the results of the inflammatory parameters, the cumulative number of pts who achieved normal body temperature (<38°C) over time was similar between regimen A and B in the first cycle, and in subsequent cycles more patients reached normal body temperature levels at earlier time points with regimen B (Figure 3A, left and center panel). This resulted in a comparable median time to achieve normal body temperatures between the groups in cycle 1 (median: 4.5d and 4d, for regimen A and B, respectively) and significantly shorter time in regimen B in subsequent cycles 2–4 (median: 5d and 1d for regimen A and B, respectively; Figure 3A, right panel). Notably, the cumulative number of pts with normal vital signs over time and the median time to achieve normal vital signs were similar between the groups (Figure 3B). Finally, most pts treated with regimen A (DB and IL-2) presented with a poor general condition in all cycles until day 4 of the treatment as reflected by a significantly longer time for pts to achieve a Lansky score above 70% compared to pts treated with regimen B (median: 7d for cycle 1, and 4d, 4d, 3.5d, and 1d for cycles 2, 3, 4, and 5 for regimen A and 3d for cycle 1 and 1d for cycles 2–5 for regimen B; Figure 3C). In conclusion, the IL-2 treatment negatively affected the general condition and body temperature, while vital signs were mostly stable and not different between the two regimens.

### Role of IL-2 for Co-medication During the Treatment With DB

To address the question, whether the therapy-related effects on the inflammatory response and vital parameters were reflected in the necessity of i.v. supportive therapy, we analyzed duration of morphine and metamizole usage as well as i.v. supportive therapy in general.

**Figure 4:**
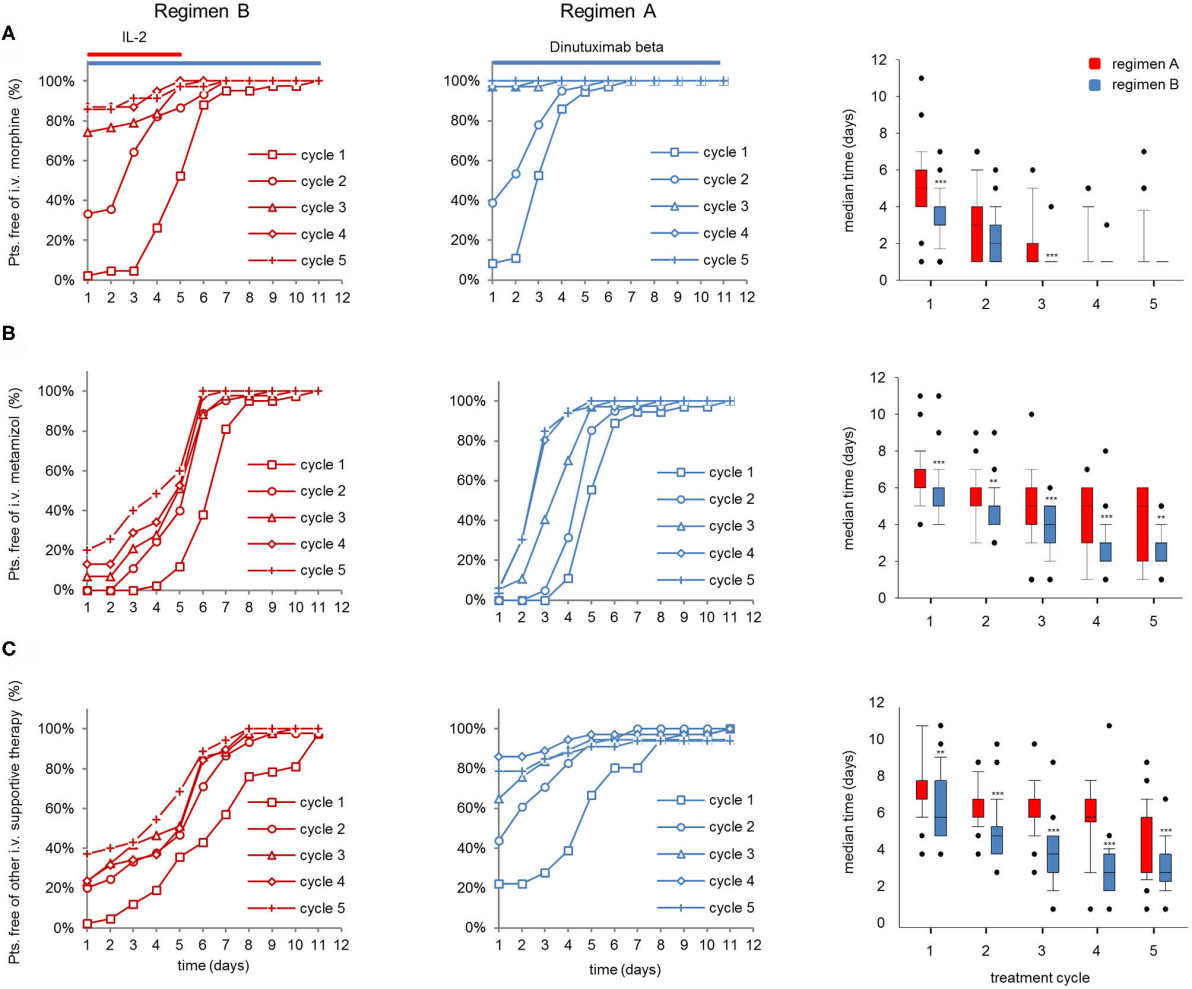
Evaluation of co-medication in patients (pts) treated with dinutuximab (DB) beta with or without s.c IL-2. Cumulative number of pts that did not need **(A)** i.v. morphine, **(B)** metamizole treatment, or **(C)** or other i.v. supportive therapy during the DB+IL-2- (left panel; *n* = 53; regimen A) and DB treatment (center; *n* = 46; regimen B) in cycle 1 (squares), cycle 2 (circles), cycle 3 (triangles), cycle 4 (diamonds), and cycle 5 (crosses) in % of the entire cohort. Comparison of median time co-medication was delivered is displayed with boxplots (right panel; **A–C**, respectively). DB LTI of the 100-mg/m^2^ (10 days) treatment is indicated with blue and the delivery of 6 × 10^6^ IU/m^2^/day IL-2 with red horizontal lines (**A–C**, right panels). Statistical analysis was performed using the Mann–Whitney-*U*-test. (**A**, right panel) ***P < 0.001 for cycles 1 and 3 vs. DB+IL-2. **(B)** ***P < 0.001 vs. DB+IL-2 for cycles 1, 3, and 4 and **P < 0.01 for cycles 2 and 5.

The improved vital parameters and lower inflammatory response with regimen B also translated into a larger proportion of pts that did not require i.v. morphine and i.v. metamizole treatment 5d after the start of the LTI compared to regimen A, particularly in the first 3 cycles (Figures 4A,B, left and center panel). In line with this, co-medication of almost all pts treated with regimen B was feasible in an “outpatient” setting starting on d5 in cycles 2–5, whereas pts treated with regimen A required intravenous co-medication requiring hospitalization of ~50% of pts in this group (Figure 4C, left and center panel). Accordingly, IL-2 treatment in combination with DB is associated with a significantly longer median time of i.v. morphine- (cycles 2 and 4) and i.v. metamizole usage as well as i.v. co-medication compared to DB treatment without IL-2 (Figures 4A–C, right panels).

In summary, pts treated with DB in combination with IL-2 needed i.v. supportive therapy for longer time periods than pts treated with DB.

### Impact of IL-2 on Time of Hospitalization and Time to Achieve Outpatient Status

Pts admitted to the hospital on d1 for the treatment with DB LTI were evaluated for time to actual discharge from the hospital and time to achieve the criterion “outpatient candidate” as described in section Patients and Methods.

**Figure 5:**
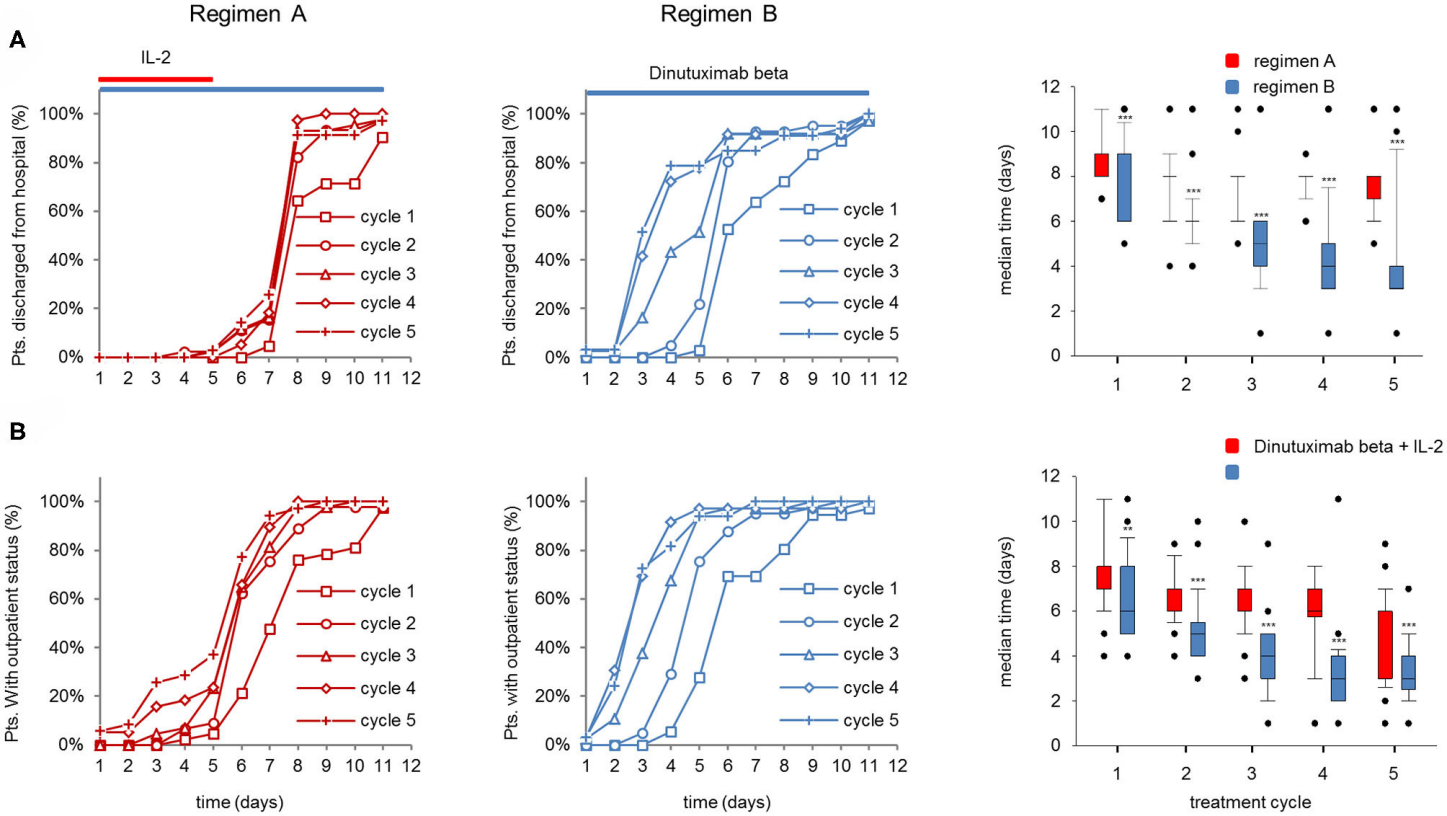
Comparison of actual discharge an potential “outpatient status” of patients (pts) treated with dinutuximab beta (DB) with or without s.c. IL-2. Time to discharge and time to achieve outpatient status (normal vital signs, good general condition, no i.v. co-medication) was analyzed on each day of the DB long-term infusion. **(A)** Time to discharge and **(B)** time needed to achieve outpatient status of pts during the DB+IL-2- (*n* = 53; left panel) (regimen A) and DB treatment (*n* = 46 center) (regimen B) in cycle 1 (squares), cycle 2 (circles), cycle 3 (triangles), cycle 4 (diamonds), and cycle 5 (crosses) in % of the entire cohort. DB LTI of 100 mg/m^2^ (10 days) treatment is indicated with blue and the delivery of 6·10^6^ IU/m^2^/day IL-2 with red horizontal lines. Boxplots (right panel) displaying median time to discharge **(A)** and to achieve potential outpatient status **(B)** in cycles 1–5 of pts treated with DB + IL-2 (red bars) or with DB alone (blue bars). Statistical analysis was performed using the Mann–Whitney-*U-*test. (**A**, right panel) ***P < 0.001 for cycles 1–5 vs. DB+IL-2. **(B)** **P < 0.001 vs. DB+IL-2 for cycle 1 and ***P < 0.001 vs. DB+IL-2 for cycles 2–5.

Notably, the majority of the pts treated with regimen A were hospitalized until d8 in all cycles, whereas the majority of pts receiving regimen B could already be discharged after 6d to 2d in cycles 1–5 (Figure 5A left and center panel). Accordingly, the median time to discharge of regimen B pts was significantly shorter compared to regimen A pts (Figure 5A, right panel, 8d and 3–6d for cycles 1–5, regimen A and B, respectively). Accordingly, the median time to achieve the “outpatient candidate” criterion was significantly shorter in regimen B compared to regimen A (Figure 5B, right panel). In pts treated with regimen A, the “outpatient candidate” status was achieved 2 days earlier compared to the actual time point of discharging the pt (cycles 2–5 in regimen A; Figures 5A,B right panel), whereas the differences between the median times in regimen A pts were similar (Figures 5A,B, right panel).

In summary, the improved treatment tolerance of regimen B compared to regimen A is reflected by an earlier time point of discharging the pt from the hospital and of achieving an “outpatient candidate” status.

## Discussion

The introduction of GD2-directed immunotherapy with DB in combination with IL-2 translated into a clear survival benefit for pts with high-risk NB (1). In these trials, DB was given as short-term infusion (20 mg/m^2^/d; 8 h; 5 consecutive days, 100 mg/m^2^/cycle), and this schedule was associated with a high rate of grade 3–4 toxicity. The induction of neuropathic pain, which can be attributed to the binding of GD2 expressed on sensory nerve fibers by anti-GD2 antibodies [off tumor on target (GD2) side effect], was observed in up to 26% of treated patients. Also, grade 3&4 inflammatory toxicity such as high fever, hypersensitivity reaction, capillary leak, and impaired general condition occurred in 40, 20, 15, and 41% of treated patients, respectively (8). Therefore, the delivery method of DB was changed from short-term to long-term continuous infusion (LTI) using 100 mg/m^2^ per cycle over 10 days in combination with the same dose and schedule of IL-2 (6). This has improved treatment tolerance and in particular the pain toxicity. However, the IL-2 treatment component continued to cause severe inflammatory side effects with a rate of 13% of treated patients developing grade 3&4 capillary leak syndrome (6, 8). The first risk benefit assessment of IL-2 co-medication with DB in the maintenance treatment of high-risk NB was reported from a prospective randomized trial (HR-NBL1/SIOPEN) (8). No evidence was found that addition of IL-2 to DB given as an 8-h short-term infusion improved outcomes in patients with high-risk neuroblastoma. Based on this landmark observation, we started to evaluate the treatment tolerance of DB without IL-2 using the long-term infusion schedule in a compassionate use program. Here, we demonstrated that DB long-term infusion without IL-2 co-medication shows a substantially improved treatment tolerance. In all analyzed parameters (clinical and laboratory), we found a beneficial safety profile for pts only treated with DB (Figures 2–4), which allowed to discharge the pts between 2 and 6 days earlier from the hospital (Figure 5), also reflecting a remarkable impact on quality of life for treated pts.

One interesting observation is also the decrease of i.v. morphine usage by omitting IL-2 (Figure 4), suggesting that there is a lower pain toxicity profile in patients treated with DB only. Due to the ability of DB to fix complement, the activation of the classical pathway of the complement system has been suggested as one of the mechanisms of pain induction. In rat models, the spinal administration of soluble complement receptor 1, which blocks the formation of complement components C3a and C5a as well as the formation of the membrane attack complex, reduced anti-GD2 antibody-mediated pain induction (13, 14). Therefore, the humanized anti-GD2 Ab hu14.18K322A with a single point mutation (K322A) abrogating complement activation was developed. In a Phase 1 study, it was shown that hu14.18K322A required lower opioid usage compared to patients treated with ch14.18 (15). However, despite the deficiency of complement activation by hu14.18K322A, pain was observed in treated pts requiring opioid co-medication, which suggests that there are other mechanisms of pain induction than CDC. The presence of a CDC-independent mechanism of pain induction by DB is also supported by our observation since the dose of DB was not different between the treatment regimens, and IL-2 does not have a direct effect on the complement system. Also, cytokines, such as IL-1 beta, IL-6, TNF, and IL-17, can induce pain by direct activation of nociceptors and cytokine blockade was effective in reducing pain (16). For example, intrathecal injection of both IL-1-beta and TNF-alpha antagonists alleviated pain induced by spinal injury and IL-6 neutralizing Ab reduced mechanical allodynia and downregulated the expression of IL-1 beta and TNF-alpha within the central nervous system (17, 18). We observed a stronger upregulation of cytokines (Figure 5) when pts received DB and IL-2 which may contribute to a higher pain toxicity profile. Consequently, the removal from IL-2 explains a lower i.v. morphine usage (Figure 4).

It is important to note that pain is also observed in the context of cytokine release syndrome induced by T-cell engaging therapies like bispecific T cell engaging (BiTE) single-chain antibody constructs and chimeric antigen receptor (CAR) T cells independent of GD2 (19). In this context, the induction of high levels of IL-6 cytokine concentrations is largely contributing to the pain induced by immunotherapies (19, 20). In line with that, we observed equally high levels of IL-6 in both cohorts suggesting that this cytokine may contribute to the observed treatment toxicity in the first cycle in both cohorts (20). Therefore, a treatment with anti-IL-6 antibodies that prevent neuropathic pain caused by IL-6 might be an option for pts that do not tolerate the Ab treatment in the first cycle (20). Although this analysis was not feasible in these single-center programs, it would be important to investigate IL-6 concentrations during subsequent cycles in future studies, particularly in pts with poor treatment 3.

The results of inflammatory parameters are in line with the improved treatment tolerance and most importantly with an earlier reduction of the i.v. morphine usage in regimen B compared to A. Considering the low inflammatory response beginning with cycle 2, a further reduction of i.v. morphine usage in pts treated only with the Ab might be possible.

We observed a steady improvement in all clinical parameters from cycle to cycle in both cohorts, which was most pronounced in the combination group treated with IL-2. We previously showed that IL-2 co-medication to DB results in a strong upregulation of CD4^+^, CD25^high^, and CD127^−^ regulatory T cells that may provide a mechanistic explanation for the alleviated inflammatory side effects in subsequent cycles (9).

In conclusion, we could show that omitting IL-2 from immunotherapy regimens with DB resulted in a clearly improved treatment tolerance, earlier discharge from the hospital and thereby improved quality of life for treated patients.

## Data Availability Statement

The raw data supporting the conclusions of this article will be made available by the authors, without undue reservation.

## Ethics Statement

The studies involving human participants were reviewed and approved by ethical committee of the University Medicine Greifswald. Written informed consent to participate in this study was provided by the participants' legal guardian/next of kin.

## Author Contributions

FC, ST-M, KC, LJ, MZ, and NS analyzed and interpreted data. FC and ST-M performed all statistical analyses. KE and HL were key contributors to the experimental concept and design. FC, ST-M, and HL were the primary author of this manuscript. All authors contributed to the writing of the manuscript and approved the final manuscript.

## Conflict of Interest

HL is a consultant of Apeiron Biologics. Apeiron Biologics had no role in the study design; in the collection, analyses or interpretation of data; in the writing of the manuscript and in the decision to publish the results. The remaining authors declare that the research was conducted in the absence of any commercial or financial relationships that could be construed as a potential conflict of interest.
